# Outcomes of Antithrombotic Therapy in Patients Undergoing Gastrointestinal Surgery: A Meta-Analysis

**DOI:** 10.7759/cureus.88473

**Published:** 2025-07-21

**Authors:** Faryal Tariq, Hasnain Ali, Abdul Moeed Baig, FNU Kajal, Rohit Raj, Sumera Afzal-Tohid, Victoria C Elochukwu Ndubuisi, Chandra Shekhar Goit

**Affiliations:** 1 General Medicine, East Kent Hospitals University NHS Foundation Trust, Margate, GBR; 2 Trauma and Orthopedics Surgery, Shifa International Hospital, Islamabad, PAK; 3 Family Medicine, Gujranwala Medical College, Gujranwala, PAK; 4 Pediatrics, Ghulam Muhammad Mahar Medical College, Sukkur, PAK; 5 Internal Medicine, Hiranand Gangabai Women Hospital, Shikarpur, PAK; 6 College of Medicine, Ziauddin Medical College, Karachi, PAK; 7 Family and Community Medicine, University of Nigeria Teaching Hospital, Enugu, NGA; 8 Internal Medicine, Kathmandu Medical College, Kathmandu, NPL

**Keywords:** antithrombotic therapy, bleeding complications, gastrointestinal surgery, perioperative outcomes, thromboembolic risk

## Abstract

Antithrombotic therapy is commonly prescribed for the prevention and management of thromboembolic diseases but poses significant challenges in the perioperative setting of gastrointestinal (GI) surgery due to the competing risks of bleeding and thrombosis. This meta-analysis aimed to evaluate the clinical outcomes associated with perioperative antithrombotic therapy in patients undergoing GI surgery. A systematic search was conducted in PubMed, Cochrane Central Register of Controlled Trials (CENTRAL), Scopus, ProQuest, and Google Scholar, following Preferred Reporting Items for Systematic Review and Meta-Analyses (PRISMA) guidelines. Nine studies comprising 13,107 patients were included. The primary outcomes assessed were postoperative bleeding, thromboembolic events, and blood transfusion requirements. Statistical analysis was performed using a DerSimonian-Laird random-effects model, and outcomes were reported as odds ratios (ORs) with 95% confidence intervals (CIs). The pooled analysis showed a non-significant trend toward increased postoperative bleeding in patients on antithrombotic therapy (OR: 1.64, 95% CI: 0.98-2.75, p = 0.061). Thromboembolic events did not differ significantly between groups (OR: 0.91, 95% CI: 0.41-2.04, p = 0.82), with low heterogeneity (I² = 0%). However, transfusion requirements were significantly higher in the antithrombotic group (OR: 2.87, 95% CI: 1.19-6.95, p = 0.019), indicating an increased need for intraoperative or postoperative blood products. While continuation of aspirin monotherapy appeared relatively safe in elective settings, dual antiplatelet therapy and direct oral anticoagulants (DOACs) were associated with greater bleeding risks, particularly in complex or minimally invasive procedures. These findings suggest that with careful patient selection and perioperative planning, antithrombotic therapy can often be safely managed during GI surgery. However, individualized risk assessment remains critical to minimize adverse outcomes. Further randomized controlled trials are needed to establish clear guidelines for antithrombotic management in this surgical population.

## Introduction and background

The management of antithrombotic therapy in patients undergoing gastrointestinal (GI) surgery presents a significant clinical dilemma [1]. Antithrombotic agents, including anticoagulants and antiplatelet medications, are widely prescribed for the prevention and treatment of thromboembolic disorders such as atrial fibrillation, venous thromboembolism, mechanical heart valves, and coronary artery disease [2]. With the global increase in the aging population and chronic cardiovascular conditions, a growing number of patients on long-term antithrombotic therapy are presenting for both elective and emergency GI surgical procedures [1,3].

The perioperative management of these agents requires a careful balance between minimizing the risk of thromboembolic complications and avoiding excessive surgical bleeding. Continuation of therapy may increase intraoperative and postoperative bleeding, while interruption -- especially without appropriate bridging -- may expose patients to life-threatening thromboembolic events [4]. The variation in clinical practice, including decisions on whether to pause, bridge, or continue therapy, reflects the absence of universally accepted guidelines, particularly in the setting of abdominal and GI surgeries, which often carry high bleeding risks [5].

Previous studies have reported conflicting outcomes. Some suggest that continuing antithrombotic therapy increases postoperative bleeding and transfusion requirements [6,7], while others report that temporary discontinuation, particularly without bridging, raises the risk of stroke, deep vein thrombosis, or pulmonary embolism [2,8,9]. The decision-making process is further complicated by the heterogeneity in surgical techniques, urgency of procedures, type and pharmacokinetics of antithrombotic agents, and patient-specific thrombotic risk profiles.

To date, no comprehensive synthesis of the evidence has clarified the balance of risks and benefits in this setting. Therefore, this meta-analysis aims to systematically evaluate and quantify the clinical outcomes associated with antithrombotic therapy in patients undergoing GI surgery. Specifically, we assess the impact of perioperative antithrombotic management strategies on postoperative bleeding, thromboembolic events, transfusion requirements, reoperations, mortality, and hospital stay. By providing pooled estimates across diverse studies, this review intends to inform perioperative decision-making and support the development of evidence-based guidelines for safe and effective management of antithrombotic therapy in GI surgical patients.

## Review

Search strategy

This meta-analysis followed the Preferred Reporting Items for Systematic Review and Meta-Analyses (PRISMA) guidelines [10]. A systematic search was conducted in the databases PubMed, Cochrane Central Register of Controlled Trials (CENTRAL), Scopus, ProQuest, and Google Scholar to identify studies examining the impact of antithrombotic therapy on outcomes in patients undergoing gastrointestinal (GI) surgery. The search covered literature published from January 2015 to March 2025 and was limited to articles published in English.

The search strategy combined Medical Subject Headings (MeSH) terms and relevant keywords, including: “Antithrombotic Therapy,” “Anticoagulants,” “Antiplatelet Agents,” “Gastrointestinal Surgery,” “Postoperative Complications,” and “Bleeding Risk.” All retrieved citations were imported into EndNote X9 (Clarivate, Philadelphia, PA) for screening and automatic deduplication.

Study selection

Two reviewers independently screened the titles and abstracts of all retrieved studies to determine eligibility for inclusion. When necessary, full-text articles were reviewed to confirm inclusion, and any discrepancies were resolved through discussion or, if needed, by consulting a third reviewer.

Studies were included if they involved adult patients undergoing any form of gastrointestinal (GI) surgery and assessed the impact of perioperative antithrombotic therapy (including anticoagulants or antiplatelet agents). Eligible studies were required to compare outcomes in patients receiving antithrombotic therapy with those who either did not receive such therapy or received a modified regimen, such as discontinuation or bridging. Additionally, studies were required to report at least one relevant clinical outcome, including postoperative bleeding, thromboembolic events, transfusion requirements, reoperation, length of hospital stay, or mortality. Both randomized controlled trials (RCTs) and observational studies (cohort and case-control designs) were considered eligible for inclusion.

Studies were excluded if they focused on pediatric populations, lacked extractable outcome data, or were limited to case reports, editorials, conference abstracts, narrative reviews, or non-English language publications. Studies that did not specifically address gastrointestinal surgical procedures or antithrombotic therapy were also excluded.

Data extraction

Two reviewers independently extracted relevant data from the included studies using a standardized data collection form. The extracted information included key study characteristics such as the first author's name, year of publication, country of origin, and study design. Patient demographic data were also collected, including age, sex, comorbid conditions, and the clinical indication for antithrombotic therapy.

Details regarding the surgical intervention were recorded, including the type of gastrointestinal surgery performed (e.g., colorectal, gastric, or hepatobiliary procedures) and whether the surgery was elective or performed under emergency conditions. The specifics of the antithrombotic therapy were documented, encompassing the type of agent used (e.g., warfarin, direct oral anticoagulants (DOACs), aspirin, or clopidogrel) and the perioperative management strategy employed, such as continuation, temporary discontinuation, or bridging therapy.

Outcome measures extracted from the studies included postoperative bleeding events (categorized as major or minor), thromboembolic complications, in-hospital or short-term mortality, reintervention or reoperation rates, transfusion requirements, and overall length of hospital stay. Any disagreements or inconsistencies in the extracted data were resolved through discussion between the two reviewers or, when necessary, by consultation with a third reviewer.

Statistical analysis and quality assessment

All statistical analyses were performed using RStudio Version 2022.02.0-443 (RStudio PBC, Boston, MA) with the "meta" and "metafor" packages. Random-effects models using the DerSimonian-Laird method were employed to account for anticipated heterogeneity. Dichotomous outcomes (e.g., bleeding events, thromboembolic complications, mortality) were analyzed using odds ratios (ORs) with 95% confidence intervals (CIs). Continuous variables (e.g., length of stay) were reported as mean differences (MDs).

Heterogeneity was assessed using the I² statistic, with values above 50% considered indicative of moderate to high heterogeneity. Publication bias was evaluated using funnel plots and Egger's test, where applicable. Sensitivity analyses were conducted by excluding studies at high risk of bias or using alternative antithrombotic strategies (e.g., bridging only).

Study quality was independently assessed using the Cochrane risk-of-bias 2 (RoB 2) tool for RCTs and the Newcastle-Ottawa Scale (NOS) for observational studies. Each study was rated as having low, moderate, or high risk of bias based on selection bias, comparability, and outcome reporting.

Summary of included studies

The initial search across selected databases and registers yielded a total of 714 records. After removing 122 duplicate records, two ineligible, and two removed for other reasons, 588 unique records remained for screening. During the title and abstract screening phase, 432 records were excluded for various reasons, such as being editorials, case reports, or not pertaining to the outcomes of antithrombotic therapy in gastrointestinal surgery. A total of 156 reports were sought for retrieval, of which seven could not be retrieved due to access limitations or unavailable full texts. The remaining 149 full-text articles were assessed for eligibility.

**Figure 1:**
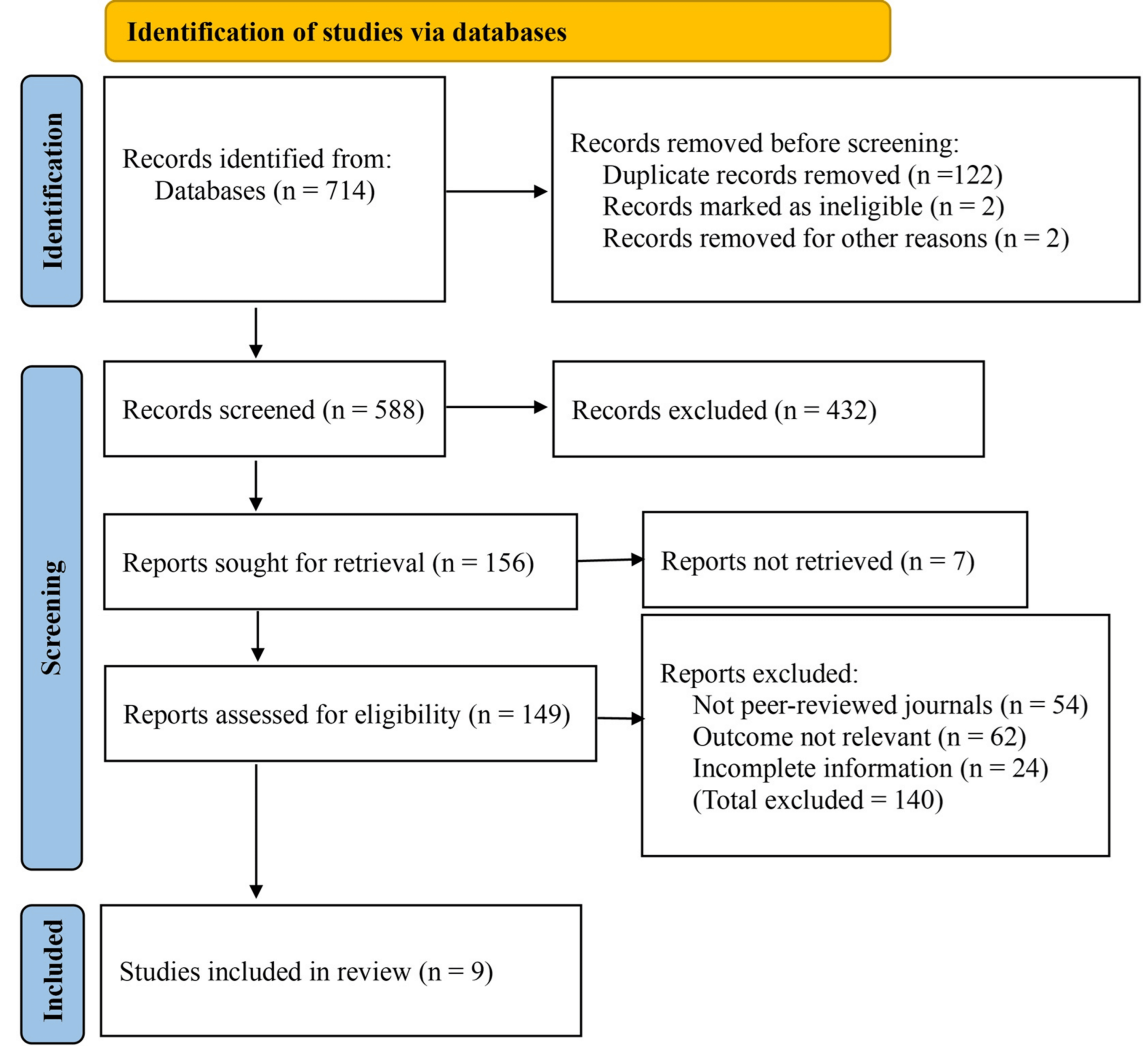
PRISMA flow diagram of study selection process. PRISMA: Preferred Reporting Items for Systematic Review and Meta-Analyses.

Following full-text review, 139 studies were excluded. These included 54 articles published in non-peer-reviewed sources, 62 studies that did not report relevant outcomes, such as bleeding or thromboembolic complications, and 24 studies with incomplete or unclear data on perioperative antithrombotic management. Ultimately, nine studies met the inclusion criteria and were included in the final meta-analysis (Figure 1).

Table 1 presents a comparative overview of nine primary studies included in this meta-analysis, each investigating the outcomes of antithrombotic therapy in patients undergoing gastrointestinal (GI) surgery. These studies span across Japan, China, South Korea, and Germany, with sample sizes ranging from 146 to over 6000 patients. The selected studies encompass a variety of GI surgical procedures, including colorectal, gastric, hepatobiliary, and emergency abdominal surgeries, and include both retrospective cohort analyses and propensity score-matched observational studies.

The majority of these studies focused on evaluating key clinical outcomes, such as intraoperative and postoperative bleeding, thromboembolic complications, blood transfusion requirements, and mortality. Several investigations specifically examined the perioperative management of aspirin and other antiplatelet agents, while others assessed the use of direct oral anticoagulants (DOACs) and vitamin K antagonists (VKAs), comparing continued versus discontinued antithrombotic regimens. For instance, studies by Takahashi et al. [11] and Yoshimoto et al. [12] analyzed colorectal cancer patients undergoing laparoscopic surgery and found no significant increase in bleeding risk with continued aspirin therapy. Similarly, Matsuoka et al. [13] and Ohya et al. [14] reported no substantial differences in surgical outcomes or bleeding rates between continuation and discontinuation groups in emergency or elective settings.

Notably, Matsui et al.'s [15] large cohort analysis on gastric cancer patients and Jang et al.'s [16] cohort study on anticoagulated patients with GI bleeding both highlighted the delicate balance between bleeding risk and thromboembolic protection. Harada et al. [17] further emphasized the increased risk of delayed bleeding in patients receiving DOACs, particularly following minimally invasive GI surgery, underscoring the importance of timing and agent-specific management strategies.

Collectively, the findings suggest that in appropriately selected patients, antithrombotic therapy, especially aspirin and certain DOACs, can often be safely continued without significantly increasing bleeding complications. However, tailored perioperative strategies remain critical, especially in high-risk patients or procedures with substantial bleeding risk. These data contribute to the growing body of evidence guiding the safe integration of antithrombotic therapy in GI surgical care.

**Table 1:** Details of selected studies. PSM: Propensity score-matched; GI: gastrointestinal; APT: antiplatelet therapy; NOACs: non-vitamin K oral anticoagulants; VKAs: vitamin K antagonists; CRC: colorectal cancer; MIS: minimally invasive surgery.

Author(s)	Country of study	Number of patients	Methodology type	Sample size	Outcomes
Takahashi and Fujikawa [11]	Japan	789	Propensity score-matched (retrospective)	210 (PSM)	Bleeding complications, transfusion needs, timing of reinstitution of antithrombotics
Abe et al. [18]	China	732	Retrospective	732	Intra/post-op bleeding, blood loss, transfusion rates, thrombotic events
Matsuoka et al. [13]	Japan	1555	Retrospective (PSM)	234 (PSM)	Intraoperative blood loss in emergency GI surgery, safety of operating while on antithrombotics
Yoshimoto et al. [12]	Japan	578	Retrospective cohort	578	Intra/post-op bleeding, thromboembolic complications, safety of continued APT in laparoscopic CRC surgery
Matsui et al. [15]	Japan	6798	Propensity matching analysis	600 (matched)	Bleeding complications, continuation vs. discontinuation of APT in gastric cancer surgery
Pannach et al. [19]	Germany	1039	Registry analysis (prospective)	1039	Types and outcomes of GI bleeding under NOAC, VKA, and APT; hospitalization and mortality rates
Ohya et al. [14]	Japan	214	Multicenter retrospective	214	Comparison of continued vs. discontinued APT; hemorrhagic and thromboembolic events
Jang et al. [16]	South Korea	146	Retrospective cohort	146	Rebleeding, thromboembolic events after OAC discontinuation vs. continuation post-GI bleed
Harada et al. [17]	Japan	1290	Retrospective cohort	1290	Postoperative bleeding, delayed bleeding, thromboembolic risk in MIS under ACT

This meta-analysis included nine primary studies comparing clinical outcomes between patients undergoing gastrointestinal (GI) surgery with and without perioperative antithrombotic therapy. Outcomes of interest included postoperative bleeding, thromboembolic complications, and the need for blood transfusion. Statistical analysis was conducted using a DerSimonian-Laird random-effects model. Dichotomous outcomes were expressed as odds ratios (ORs) with 95% confidence intervals (CIs), and significance was assessed at p < 0.05. Heterogeneity was evaluated using I² and τ² statistics, along with χ² tests.

Postoperative bleeding

**Figure 2:**
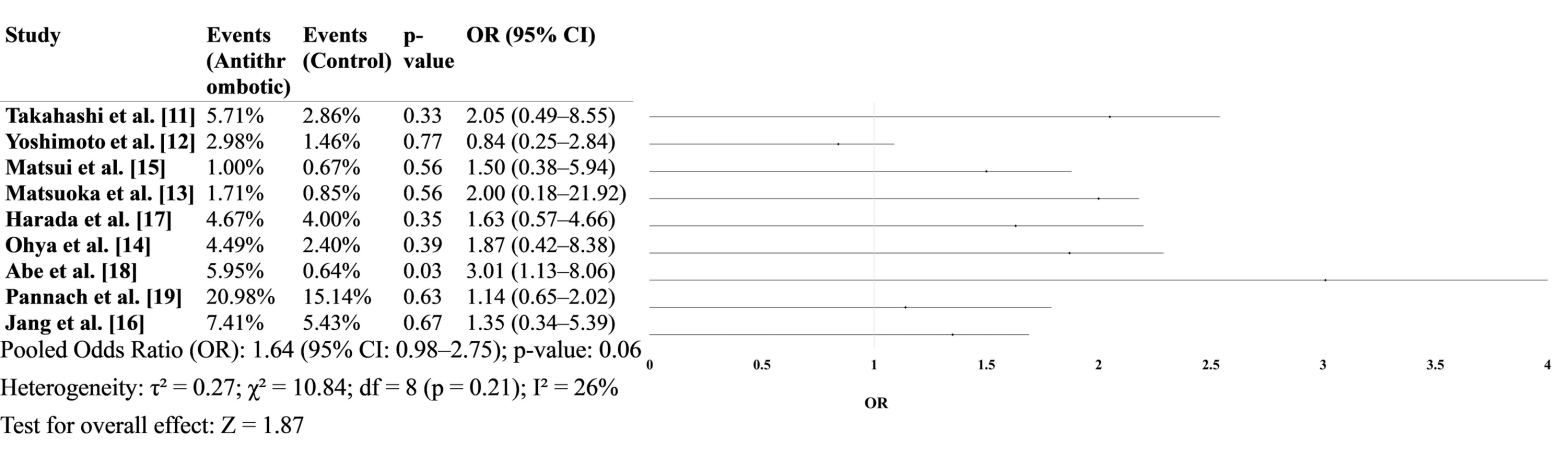
Postoperative bleeding events in antithrombotic vs. non-antithrombotic groups

A total of nine studies [11-20] reported data on postoperative bleeding events. While individual study results varied, the pooled analysis did not reach statistical significance, though a trend toward increased bleeding risk was observed in the antithrombotic group, particularly in the study by Abe et al. [18], which showed a significant association (Figure 2).

Thromboembolic events

**Figure 3:**
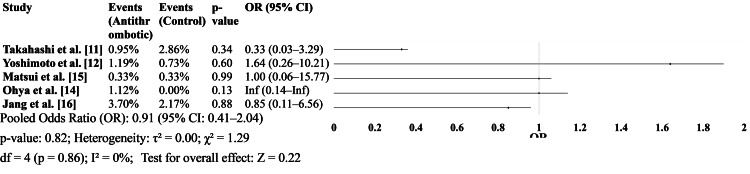
Thromboembolic events in antithrombotic vs. non-antithrombotic groups.

Five studies [11,12,14-16,18] reported data on thromboembolic events. Overall, there was no statistically significant difference between the antithrombotic and non-antithrombotic groups. Odds ratios across studies were generally close to 1.0, and heterogeneity was low, indicating consistent findings (Figure 3).

Blood transfusion requirement

**Figure 4:**
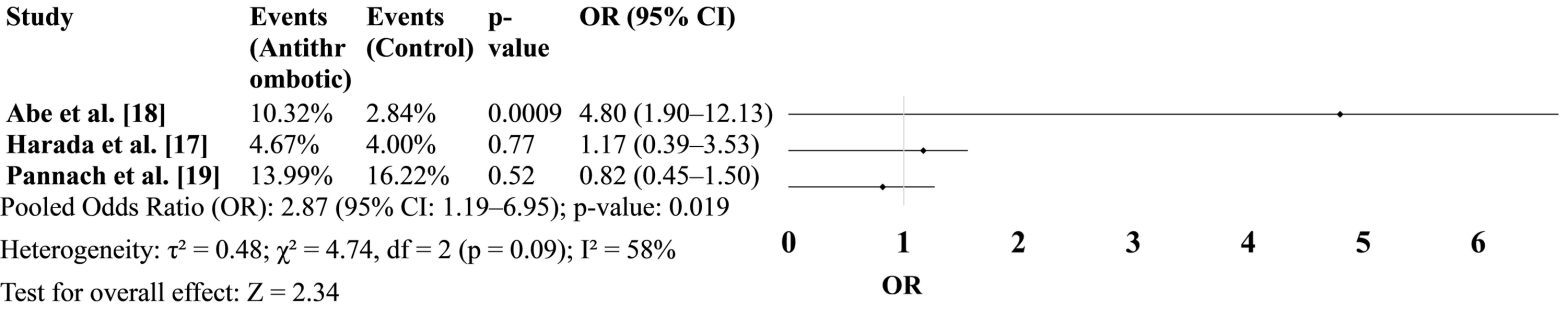
Blood transfusion in antithrombotic vs. non-antithrombotic groups.

Figure 4 reports on the need for perioperative blood transfusion in patients receiving antithrombotic therapy. One large study [20] showed a significantly higher transfusion rate in the antithrombotic group, which influenced the pooled estimate. Despite moderate heterogeneity, the overall analysis demonstrated a significant association between antithrombotic therapy and increased transfusion requirements (Figure 4).

Discussion

This meta-analysis aimed to assess the safety and clinical outcomes associated with perioperative antithrombotic therapy in patients undergoing gastrointestinal (GI) surgery. Specifically, we evaluated postoperative bleeding, thromboembolic events, and blood transfusion requirements across a diverse range of studies from East Asia and Europe. Our findings reflect a nuanced interplay between thrombotic protection and bleeding risk, with implications for perioperative decision-making in surgical patients on chronic antithrombotic regimens.

Our pooled analysis of postoperative bleeding showed a trend toward increased bleeding in patients receiving antithrombotic therapy, with a non-significant odds ratio of 1.64 (95% CI: 0.98-2.75, p = 0.061). Notably, Abe et al. [18] demonstrated a statistically significant elevation in bleeding risk (OR: 3.01, 95% CI: 1.13-8.06, p = 0.03), which heavily influenced the pooled estimate.

This trend is consistent with findings from other surgical disciplines. For instance, a recent meta-analysis by [20] found significantly increased postoperative bleeding (RR: 4.77, 95% CI: 1.13-20.10) in patients on antithrombotic therapy undergoing laparoscopic cholecystectomy [5]. Similarly, studies like those by Yoshimoto et al. [12] reported higher, but not statistically significant, bleeding rates among patients on antiplatelet therapy (APT) undergoing laparoscopic colorectal surgery, suggesting that the risk may be context-dependent and modulated by surgical technique and perioperative protocols.

Interestingly, Matsuoka et al. [13] found no significant association between antithrombotic drug use and bleeding outcomes after adjusting for confounders via propensity score matching, indicating that observed bleeding may be more attributable to baseline patient risk than the medications themselves. Furthermore, Matsui et al. [15] noted no significant difference in bleeding complications between continuation and discontinuation groups in gastric cancer surgery, reinforcing the safety of continued antithrombotic use in well-selected cases.

Our analysis of thromboembolic events revealed no significant difference between antithrombotic and non-antithrombotic groups (OR: 0.91, 95% CI: 0.41-2.04, p = 0.82), with minimal heterogeneity (I² = 0%). These findings are critical as they support the notion that withholding antithrombotic therapy may not offer substantial benefit in terms of reducing thromboembolic risk, and conversely, continued therapy does not confer excessive thrombotic risk either.

Yoshimoto et al. [12] emphasized that thromboembolic complications, although less frequent than bleeding events, were more likely to result in severe outcomes, such as myocardial infarction or stroke, which warrants cautious perioperative planning, particularly in patients with high cardiovascular risk profiles. This aligns with guidelines recommending the continuation of aspirin monotherapy in patients at high thromboembolic risk undergoing non-cardiac surgery [21,22].

Moreover, in our analysis, all included studies consistently reported low thromboembolic event rates. This may reflect the efficacy of modern perioperative thromboprophylaxis protocols and improvements in patient optimization, particularly in minimally invasive surgical settings.

We observed a statistically significant increase in perioperative blood transfusion among patients receiving antithrombotic therapy (OR: 2.87, 95% CI: 1.19-6.95, p = 0.019). This result was primarily driven by the study of Abe et al. [18], which reported a significantly higher transfusion rate in the antithrombotic group (OR: 4.80, p = 0.0009) [18]. These findings suggest that while major bleeding events may not always occur, minor or subclinical bleeding necessitating transfusion may still be more common in patients receiving antithrombotics.

A similar conclusion was drawn in the analysis by Matsuoka et al. [13], where pre-matching data showed higher transfusion rates and blood loss in patients on antithrombotics, although post-matching comparisons found no significant differences. This disparity underscores the influence of patient selection and comorbidities on transfusion requirements, suggesting that transfusion risk may be modifiable through individualized surgical planning and conservative transfusion thresholds.

In contrast, other studies, such as that by Matsui et al. [15], found no significant difference in transfusion rates after propensity score matching, reaffirming the importance of controlling for confounders. Additionally, Fujikawa et al. [23] found that non-vitamin K oral anticoagulants (NOACs) but not aspirin were associated with increased bleeding complications, suggesting agent-specific differences in transfusion risk.

Clinical implications

Collectively, these findings support a risk-stratified approach to perioperative antithrombotic management. While there is a trend toward increased bleeding and transfusion risk, this does not universally translate into clinically significant adverse outcomes. Importantly, the continuation of aspirin monotherapy, especially in laparoscopic or low-risk procedures, appears to be safe and may even be preferable to avoid thrombotic events in high-risk populations, as noted in prior literature [24-26].

The low incidence of thromboembolic complications further suggests that careful perioperative planning, including optimal timing for drug discontinuation and resumption, as well as utilization of bridging protocols when needed, can mitigate risks effectively. However, caution is warranted with NOACs, which have been shown to have a stronger association with bleeding complications in both our study and previous findings [24,27].

Limitations

Several limitations should be considered. Despite the use of propensity score matching in some included studies, inherent biases associated with retrospective designs remain. Variations in surgical techniques, perioperative protocols, and definitions of bleeding across studies may contribute to heterogeneity. Furthermore, the influence of dual antiplatelet therapy (DAPT) and specific anticoagulants, such as NOACs or VKAs, could not be separately evaluated in all cases due to limited subgroup data.

## Conclusions

This meta-analysis suggests that perioperative continuation of antithrombotic therapy, particularly aspirin, does not significantly increase the risk of major postoperative bleeding or thromboembolic events in patients undergoing gastrointestinal surgery. However, there is a modest increase in transfusion requirements. These findings support individualized perioperative management strategies that balance thrombotic and bleeding risks based on patient comorbidities, surgical context, and antithrombotic regimen.

## References

[1] Talasaz AH, Sadeghipour P, Ortega-Paz L (2024). Optimizing antithrombotic therapy in patients with coexisting cardiovascular and gastrointestinal disease. Nat Rev Cardiol.

[2] Kumbhani DJ, Cannon CP, Beavers CJ (2021). 2020 ACC expert consensus decision pathway for anticoagulant and antiplatelet therapy in patients with atrial fibrillation or venous thromboembolism undergoing percutaneous coronary intervention or with atherosclerotic cardiovascular disease: a report of the American College of Cardiology Solution Set Oversight Committee. J Am Coll Cardiol.

[3] Fanaroff AC, Li S, Marquis-Gravel G (2021). Atrial fibrillation and coronary artery disease: a long-term perspective on the need for combined antithrombotic therapy. Circ Cardiovasc Interv.

[4] Moster M, Bolliger D (2022). Perioperative guidelines on antiplatelet and anticoagulant agents: 2022 update. Curr Anesthesiol Rep.

[5] Sengupta N, Feuerstein JD, Jairath V, Shergill AK, Strate LL, Wong RJ, Wan D (2023). Management of patients with acute lower gastrointestinal bleeding: an updated ACG guideline. Am J Gastroenterol.

[6] Atar D, Vandenbriele C, Agewall S (2025). Management of patients with congenital bleeding disorders and cardiac indications for antithrombotic therapy: a clinical consensus statement of the ESC Working Group on Thrombosis, the Association for Acute CardioVascular Care (ACVC), European Association of Percutaneous Cardiovascular Interventions (EAPCI), European Heart Rhythm Association (EHRA) of the ESC, the ESC Working Group on Cardiovascular Pharmacotherapy and the European Association for Haemophilia and Allied Disorders (EAHAD). Eur Heart J Cardiovasc Pharmacother.

[7] Shah S, Urtecho M, Firwana M (2022). Perioperative management of antiplatelet therapy: a systematic review and meta-analysis. Mayo Clin Proc Innov Qual Outcomes.

[8] Gorog DA, Gue YX, Chao TF (2022). Assessment and mitigation of bleeding risk in atrial fibrillation and venous thromboembolism: executive summary of a European and Asia-Pacific expert consensus paper. Thromb Haemost.

[9] Gorog DA, Gue YX, Chao TF (2022). Assessment and mitigation of bleeding risk in atrial fibrillation and venous thromboembolism: a Position Paper from the ESC Working Group on Thrombosis, in collaboration with the European Heart Rhythm Association, the Association for Acute CardioVascular Care and the Asia-Pacific Heart Rhythm Society. Europace.

[10] O'Dea RE, Lagisz M, Jennions MD (2021). Preferred reporting items for systematic reviews and meta-analyses in ecology and evolutionary biology: a PRISMA extension. Biol Rev Camb Philos Soc.

[11] Takahashi R, Fujikawa T (2021). Impact of perioperative aspirin continuation on bleeding complications in laparoscopic colorectal cancer surgery: a propensity score-matched analysis. Surg Endosc.

[12] Yoshimoto Y, Fujikawa T, Tanaka A (2019). Optimal use of antiplatelet agents, especially aspirin, in the perioperative management of colorectal cancer patients undergoing laparoscopic colorectal resection. World J Surg Oncol.

[13] Matsuoka T, Kobayashi K, Lefor AK, Sasaki J, Shinozaki H (2019). Antithrombotic drugs do not increase intraoperative blood loss in emergency gastrointestinal surgery: a single-institution propensity score analysis. World J Emerg Surg.

[14] Ohya H, Watanabe J, Suwa Y (2021). Comparison of the continuation and discontinuation of perioperative antiplatelet therapy in laparoscopic surgery for colorectal cancer: a retrospective, multicenter, observational study (YCOG 1603). Ann Gastroenterol Surg.

[15] Matsui R, Nunobe S, Makuuchi R (2023). Relationship between antithrombotic drugs and postoperative outcomes in patients with gastric cancer after gastrectomy: a propensity matching analysis. Gastric Cancer.

[16] Jang HJ, Lee D, Kim TH, Kim JS, Lee HJ, Kim JB, Kim JY (2022). Clinical outcomes of gastrointestinal bleeding management during anticoagulation therapy. PLoS One.

[17] Harada K, Uemoto Y, Nagata K (2025). The effect of anticoagulation therapy on the surgical outcomes of minimally invasive major gastrointestinal surgery. Surg Endosc.

[18] Abe S, Ami K, Katsuno A (2021). Emergency gastrointestinal surgery in patients undergoing antithrombotic therapy in a single general hospital: a propensity score-matched analysis. BMC Gastroenterol.

[19] Pannach S, Goetze J, Marten S, Schreier T, Tittl L, Beyer-Westendorf J (2017). Management and outcome of gastrointestinal bleeding in patients taking oral anticoagulants or antiplatelet drugs. J Gastroenterol.

[20] Cai Y, Zhang J, Chen F (2024). The impact of anti-thrombotic therapy on bleeding outcomes and thrombosis following laparoscopic cholecystectomy: a meta-analysis. Updates Surg.

[21] Chan FK, Goh KL, Reddy N (2018). Management of patients on antithrombotic agents undergoing emergency and elective endoscopy: joint Asian Pacific Association of Gastroenterology (APAGE) and Asian Pacific Society for Digestive Endoscopy (APSDE) practice guidelines. Gut.

[22] Fujimoto K, Fujishiro M, Kato M (2014). Guidelines for gastroenterological endoscopy in patients undergoing antithrombotic treatment. Dig Endosc.

[23] Fujikawa T, Tanaka A, Abe T, Yoshimoto Y, Tada S, Maekawa H, Shimoike N (2013). Does antiplatelet therapy affect outcomes of patients receiving abdominal laparoscopic surgery? Lessons from more than 1,000 laparoscopic operations in a single tertiary referral hospital. J Am Coll Surg.

[24] Sueta D, Tabata N, Ikeda S (2019). Differential predictive factors for cardiovascular events in patients with or without cancer history. Medicine (Baltimore).

[25] Mita K, Ito H, Murabayashi R (2012). Postoperative bleeding complications after gastric cancer surgery in patients receiving anticoagulation and/or antiplatelet agents. Ann Surg Oncol.

[26] Katai H, Mizusawa J, Katayama H (2017). Short-term surgical outcomes from a phase III study of laparoscopy-assisted versus open distal gastrectomy with nodal dissection for clinical stage IA/IB gastric cancer: Japan Clinical Oncology Group Study JCOG0912. Gastric Cancer.

[27] Fujikawa T, Takahashi R, Naito S (2020). Perioperative antithrombotic management of patients who receive direct oral anticoagulants during gastroenterological surgery. Ann Gastroenterol Surg.

